# Syllabus of Lectures on Human Embryology

**Published:** 1907-04

**Authors:** 


**Syllabus of Lectures on Human Embryology.** With a Glossary of Embryological Terms. By **Walter Porter Manton,** M.D., Professor of Clinical Gynecology and Professor adjunct of Obstetrics in the Detroit College of Medicine. Third edition. Illustrated. 12mo., pp. 136. Philadelphia: F. A. Davis Co. 1906.

In addition to a thorough revision of the text for this third edition of a very helpful volume, much new material has been incorporated, and as an elementary basis for the study of the advanced branches of the specialties of obstetrics and gynecology it has a place in medical literature which it fills with much credit to its author. Especially good are the chapters on practical work in embryology and the methods of laboratory instruction. Many new illustrations have been added and the pictorial feature is one which gives value to the work and helps to make it more understandable and instructive. N. W. W.

